# Molecular Detection of *Borrelia* Bacteria in Cerebrospinal Fluid-Optimisation of Pre-Analytical Sample Handling for Increased Analytical Sensitivity

**DOI:** 10.3390/diagnostics11112088

**Published:** 2021-11-12

**Authors:** Malin Lager, Peter Wilhelmsson, Andreas Matussek, Per-Eric Lindgren, Anna J. Henningsson

**Affiliations:** 1 Division of Clinical Microbiology, Laboratory Medicine (Region Jönköping County, 553 05 Jönköping, Sweden), Department of Biomedical and Clinical Sciences, Linköping University, 581 83 Linköping, Sweden; peter.wilhelmsson@liu.se (P.W.); anmatu@ous-hf.no (A.M.); per-eric.lindgren@liu.se (P.-E.L.); anna.jonsson.henningsson@rjl.se (A.J.H.); 2 Division of Inflammation and Infection, Department of Biomedical and Clinical Sciences, Linköping University, 581 83 Linköping, Sweden; 3 Division of Laboratory Medicine, Oslo University Hospital, 0450 Oslo, Norway; 4 Institute of Clinical Medicine, University of Oslo, 0316 Oslo, Norway; 5 Division of Clinical Microbiology, Department of Biomedical and Clinical Sciences, Linköping University, 581 83 Linköping, Sweden

**Keywords:** Lyme borreliosis, Lyme neuroborreliosis, polymerase chain reaction (PCR), cerebrospinal fluid, clinical diagnostic, optimization

## Abstract

The main tools for clinical diagnostics of Lyme neuroborreliosis (LNB) are based on serology, i.e., detection of antibodies in cerebrospinal fluid (CSF). In some cases, PCR may be used as a supplement, e.g., on CSF from patients with early LNB. Standardisation of the molecular methods and systematic evaluation of the pre-analytical handling is lacking. To increase the analytical sensitivity for detection of *Borrelia* bacteria in CSF by PCR targeting the *16S* rRNA gene, parameters were systematically evaluated on CSF samples spiked with a known amount of cultured *Borrelia* bacteria. The results showed that the parameters such as centrifugation time and speed, the use of complementary DNA as a template (in combination with primers and a probe aiming at target gene *16S* rRNA), and the absence of inhibitors (e.g., erythrocytes) had the highest impact on the analytical sensitivity. Based on these results, a protocol for optimised handling of CSF samples before molecular analysis was proposed. However, no clinical evaluation of the proposed protocol has been done so far, and further investigations of the diagnostic sensitivity need to be performed on well-characterised clinical samples from patients with LNB.

## 1. Introduction

Lyme neuroborreliosis (LNB) is a disease caused by tick-borne *Borrelia* bacteria and constitutes about 3–12% of all borreliosis cases in Europe and in the USA [1]. LNB is divided into early and late LNB, and >95% of all cases are categorised as early LNB, i.e., diagnosed within six months from the onset of symptoms. The most common clinical manifestations of LNB in Europe are lymphocytic meningitis, facial palsy, and radicular pain (Bannwarth’s syndrome). Diagnosis of LNB is based on the patient´s medical history and clinical signs and symptoms together with leucocytosis in the cerebrospinal fluid (CSF) and an elevated anti-*Borrelia* antibody index as an indication of intrathecal production of specific antibodies [2]. Other *Borrelia* species, like the relapsing fever species *Borrelia miyamotoi*, can also cause systemic illness in humans. The *B. miyamotoi* bacteria can be detected in both serum and CSF by serological or molecular analysis [3,4]. However, *B. miyamotoi* infection is rarely detected in serological assays used for detection of *Borrelia burgdorferi* sensu lato (s.l.), and specific serological tests are not commercially available. Instead, in cases of suspected *B. miyamotoi* disease, PCR can be used as a diagnostic tool since the diagnostic sensitivity is high in both serum, plasma, and CSF [5,6,7].

Currently, the methods for laboratory diagnosis of LNB consist mainly of serological tests, like enzyme-linked-immunosorbent assays and immunoblot, with antibody detection in serum and CSF. Even though there are several commercial diagnostic kits available, which are well-established and frequently used in clinical practice, limitations due to cross-reactivity, delay of antibody formation, and persistence of antibodies after clearance of the infection exist. High seroprevalence in the healthy population may also hamper interpretation of serological results [8,9,10,11,12,13]. In some cases, serological analyses need to be supplemented by other diagnostic tools such as PCR. However, PCR has shown low sensitivity in CSF (median 10–30%), and it has been proposed that this may be a result of a low number of spirochetes in this sample material [2,14]. Due to the low diagnostic sensitivity, PCR is not a suitable primary analysis of *Borrelia* spp. in CSF in case of suspected LNB. However, for certain conditions like in the early LNB phase, when the antibodies have not yet been developed, PCR-based methods may serve as a supplement. For detection of *B. miyamotoi*, PCR has higher diagnostic sensitivity and is the primary diagnostic method since no commercial serological assays are currently available [4,15].

Previous studies using PCR for molecular detection of *B. burgdorferi* s.l. have mainly focused on the evaluation and comparison of different molecular protocols including different target genes and detection methods [14,16], and very few studies have compared and evaluated the handling of samples before molecular analysis (the pre-analytical procedures/handling). However, the pre-analytical procedures before PCR analysis are fundamental, especially in samples with low bacterial concentration, and suboptimal pre-analytic protocols are likely to limit the overall test performance. In some studies, the impact of storage temperature has been investigated in spiked samples or patient samples [17,18]. However, little is documented regarding the handling of samples prior to molecular analysis such as centrifugation time and speed, sample volume, type of template, and potential PCR inhibitors (e.g., erythrocytes) and how they affect the diagnostic sensitivity.

In the current situation, there is a need of standardisation for both PCR analysis and pre-analytical handling [14], and, to our knowledge, no systematic evaluation of the pre-analytical procedures has been published so far. In a previous study by Lager et al. (2017) [16], we have shown that the analytical sensitivities, specificities, and concordance among eight different PCR protocols used in laboratories in Scandinavia are high. This indicates that the protocols are well designed and evaluated and that the problem with low diagnostic sensitivity is not correlated to the protocols per se but could be, at least in part, a result of sub-optimal pre-analytical procedures before PCR analysis.

The main objective of this study was to investigate the pre-analytical handling of CSF samples before molecular testing of *Borrelia* spp. to increase the analytical sensitivity and to establish an optimised protocol for pre-analytical sample handling. This was done by systematic investigation of different parameters with a potential impact on overall test performance, such as time and speed of centrifugation, sample volume, type of template, sample storage and transportation conditions, and occurrence of leucocytes or erythrocytes in the CSF sample.

## 2. Materials and Methods

### 2.1. Experimental Setups

The study contained nine experimental setups (Table 1, Supplementary Materials SI), which will be referred to as setup/setups further on in the article. The setups were established to compare the analytical sensitivity in CSF samples spiked with known amounts of cultured *Borrelia garinii* Lu59 between:

1. Supernatant and pelleted material.
2. Different centrifugation time and speed.
3. Extraction of deoxyribonucleic acid (DNA) and extraction of total nucleic acid (NA) and, consequently, the use of DNA versus complementary DNA (cDNA) as a template.
4. Different sample volumes.
5. Different storage and transportation conditions.
6. CSF with leucocytosis and CSF without leucocytosis.
7. CSF with erythrocytes and CSF without erythrocytes.

To evaluate the reproducibility and repeatability of the optimised pre-analytical steps, established in setups I–VII, two evaluations were performed: (1) analysis of the reproducibility between different real-time PCR runs by analysing the same set of samples in two separate runs and (2) analysis of the repeatability for one sample by analysing the same sample applied in eight wells in one real-time PCR run (setup VIII).

Finally, as setup IX, the optimised pre-analytical protocol from setups I–VII was applied to CSF samples spiked with three cultured *B. burgdorferi* s.l. strains (*Borrelia afzelii* Lu81, *B. garinii* Lu59, and *B. burgdorferi* sensu stricto (s.s.) B31) and one *B. miyamotoi* strain (*B. miyamotoi* HT31). For detailed information regarding each setup, see Table 1 and Supplementary Materials SI.

### 2.2. Collection of Cerebrospinal Fluid Samples

To optimise the pre-analytical steps, anonymised CSF samples both with and without leucocytosis (defined as mononuclear cell count >5 × 10^6*/L) were collected from patients under investigation for other diseases not related to tick-borne infections. The samples were collected at the Division of Clinical Chemistry, Laboratory Medicine, Region Jönköping County, Jönköping, Sweden (2019), and all samples were pooled together (one pool for samples with leucocytosis and one without). Samples containing high levels of erythrocytes (>6 × 10^6/L) and samples from patients with clinically suspected LNB were excluded from the study. The pooled CSF samples were stored at −80 °C until spiked with *Borrelia* bacteria.

### 2.3. Culture, Bacterial Count, and Spiking of Cerebrospinal Fluid with Borrelia Strains

Clinical isolates of *B. burgdorferi* s.l. (*B. afzelii* Lu81, *B. garinii* Lu59, and *B. burgdorferi* s. s. B31) were cultured in Barbour-Stoenner-Kelly II medium [19], supplemented with 6% rabbit serum (Sigma Aldrich, St. Louis, Missouri, US). The strains were cultured at 35–37 °C for 5–7 days depending on the strain [16]. All strains were kindly provided by Sven Bergström, Umeå University, Umeå, Sweden. The *B. miyamotoi* strain HT31 was cultured at 37 °C for 5 days in modified Kelly–Pettenkofer medium [20] with fetal calf serum (pH 7.5). The culture medium and the strain were kindly provided by Barbara Johnson, Centers for Disease Control and Prevention, USA through Joppe W. Hovius, Center for Experimental and Molecular Medicine, Amsterdam Multidisciplinary Lyme Borreliosis Center, University Medical Center, Academic Medical Center, University of Amsterdam, Amsterdam, the Netherlands.

The spirochetes in the *Borrelia* cultures were checked for viability and counted repeatedly in phase-contrast microscope as previously described by Lager et al. (2017) [16]. The numbers of spirochetes per µL were based on a mean value of two counts per sample.

Before spiking, a CSF pool was thawed from storage at −80 °C. The CSF samples were spiked with cultured bacteria in a dilution series ranging from 2 × 10^−1 to 2 × 10^3 cells μL^−1^, which resulted in a final concentration of 10^0 to 10^4 cells per sample before extraction when 5 µL per dilution was used to spike 1 mL CSF (except for setup IV and V). For more details regarding *Borrelia* species, concentration of bacteria, sample volume, sample storage, CSF with or without leucocytosis, and CSF with or without erythrocytes for each setup, see Table 1 and Supplementary Materials SI.

### 2.4. Extraction of Nucleic Acid and Synthesis of Complementary DNA

The samples were in most setups in the study centrifuged at 3000× *g* for 10 min. For more details regarding centrifugation and the use of supernatant versus pelleted material for each setup, see Table 1 and Supplementary Materials SI. To each vial, 20 µL proteinase K (20 mg/mL) (Qiagen, Hilden, Germany) was added, and the samples were mixed on a vortex (Vortex-Genie^®^2, Scientific Industries, Inc., Bohemia, NY, USA) and lysed at 56 °C for 1 h before extraction of NA. For extraction of total NA, the EZ1 RNA Tissue Mini Kit (Qiagen) was used, while the EZ1 DNA Tissue Mini Kit (Qiagen) was used for extraction for DNA. Both extractions methods were performed according to the manufacturer´s instructions, and all extractions were performed on the EZ1 Advanced XL instrument (Qiagen), with an elution volume of 50 µL. After extraction, the total NA was reverse-transcribed to cDNA by using Illustra™ Ready-to-Go RT-PCR beads kit (GE Healthcare Life Science, Chicago, IL, USA, as previously described by Lager et al. (2017) [16]. For more details regarding extraction methods and template type for each setup, see Table 1 and Supplementary Materials SI. In most of the setups, NA were extracted in duplicates for each dilution, and each extraction was placed in two wells in the real-time PCR analysis. This resulted in a total of four quantification cycle (Cq)-values (one per well), which were used to calculate a mean Cq value for each dilution. For the setup comparing the storage and transportation conditions (setup V), evaluations were performed on different occasions with different sample sets.

In the setup analysing the reproducibility (setup VIII), a comparison between two separate real-time PCR runs was performed. By applying twelve samples from setup V in two separate real-time PCR runs, the difference in Cq value between the two runs could be calculated. In the setup analysing repeatability (setup VIII), one single sample (dilution 10^3 cells per sample before extraction stored in refrigerator from setup V) was applied into eight wells, and the difference in Cq values between the different wells in one PCR run was calculated.

### 2.5. Detection of Borrelia spp. by a Genus-Specific Real-Time PCR (Setups I–IX)

The changes in the analytical sensitivity of *Borrelia* spp. cells were measured by an in-house genus-specific *Borrelia* spp. *16S* rDNA real-time PCR using a CFX96™ real-time PCR detection system (Bio-Rad Laboratories, Inc., Hercules, CA) published by Gyllemark et al. (2021) [21]. The optimised conditions in a final volume of 20 µL were: Maxima Probe qPCR Mix (Thermo Fisher Scientific, Waltham, MA), 200 nM Borrelia-F primer (5’-GCT GAG TCA CGA AAG CGT AG-3’) (Thermo Fisher Scientific), 200 nM Borrelia-R primer (5’-CAC TTA ACA CGT TAG CTT CGG TA-3’) (Thermo Fisher Scientific), 200 nM Borrelia-p probe (5’-FAM-CGC TGT AAA CGA TGC ACA CTT GGT-MGB-3’) (Thermo Fisher Scientific), 5 µL of template cDNA/DNA, and RNase-free water (GE Healthcare Life Science) up to 20 µL. The cycling conditions were: 95 °C for 5 min followed by 50 cycles of 95 °C for 10 s and 60 °C for 60 s. The primers and the probe were designed in silico to detect all known *Borrelia* species. All samples were analysed as duplicates.

### 2.6. Data Analysis

For the continuous variables, the paired t-test was used to assess the significance of the differences between mean Cq values. All statistical analyses were evaluated at a significant level of 0.05, and the 95% binomial confidence interval (CI) was used. The analyses were performed using GraphPad Prism version 6.00 for Windows (GraphPad Software, La Jolla, CA, USA, www.graphpad.com, 14 April 2020).

## 3. Results

### 3.1. Pre-Analytical Handling for Increased Analytical Sensitivity

#### 3.1.1. Supernatant versus Pelleted Material (I)

The results from the comparison between use of the supernatant versus use of the pelleted material showed higher analytical sensitivity for the use of pelleted material (Table 2).

The difference in mean Cq value between extracts from the pelleted material and extracts from the supernatant was at least eight cycles, which theoretically is a >100 times higher analytical sensitivity (*p* = 0.0082) for the extracts from pelleted materials. In the extracts of the supernatants, the real-time PCR was able to detect down to 10^3 cells per sample before extraction in all samples, compared to the extracts of the pelleted material where detection down to 10^2 cells per sample before extraction in all samples was shown.

#### 3.1.2. Centrifugation Time and Speed (II)

In the comparison between different centrifugation times and speeds, centrifugation at 10,000× *g* for 60 min and centrifugation at 3000× *g* for 10 min presented comparable results down to 10^1 cells per sample (concentration before extraction) in all samples, with no major difference in analytical sensitivity (Table 3).

By use of the centrifugation speed at 10,000× *g* and the time of 60 min, the real-time PCR was able to detect down to 10^0 cells per sample (concentration before extraction) in one out of four samples (Table 3). Centrifugation at 18,840× *g* (maximum speed) for 60 min generated the highest mean Cq values by real-time PCR and detected down to 10^2 cells per sample (before extraction) in all four wells (Table 3). The mean Cq values for centrifugation at 500× *g* for 5 min showed comparable results with no major difference in analytical sensitivity to both centrifugations at 10,000× *g* for 60 min and centrifugation at 3000× *g* for 10 min down to 10^2 cells per sample (concentration before extraction) (Table 3). However, only one of four wells was positive in the detection of 10^1 cells per sample before extraction, showing that the analytical sensitivity for the low time and speed is slightly lower in samples with few spirochetes.

#### 3.1.3. DNA versus Complementary DNA as Template (III)

The results from the evaluation of the analytical sensitivity between different types of templates (cDNA and DNA) showed a higher analytical sensitivity for use of cDNA as template (Table 4).

#### 3.1.4. Sample Volumes (IV)

The comparison between the different sample volumes showed large differences between the use of 0.3 mL compared to 2.0 mL (difference = 2.50 cycles), with an advantage for 2.0 mL. However, the difference between 1.0 and 2.0 mL was less than one cycle (difference = 0.60 cycles), which makes them comparable. In general, the difference between the four volumes evaluated was about one cycle or less per sample volume: (1) volume 0.3–0.5 mL (difference = 0.90), (2) 0.5–1.0 mL (difference = 1.00), and (3) 1.0–2.0 mL (difference = 0.60) (Table 5). The results show that there was no gain in using a larger volume of CSF to increase analytical sensitivity. Based on these results, the sample volume should preferably be between 0.5–1.0 mL, since 2.0 mL did not increase the analytical sensitivity compared to 1.0 mL, and the difference in Cq value between 1.0 mL (recommended maximum volume) and 0.5 mL (recommended minimum volume) was insignificant.

#### 3.1.5. Storage and Transportation Conditions (V)

For the investigation of storage conditions, six different sets of time (days) and three different temperatures were evaluated, while the transportation conditions were evaluated at one temperature at four sets of time (days) (Table 1, Supplementary Materials SI). The storage and transportation conditions were evaluated on separate sample sets and at separate occasions. The results showed that the analytical sensitivity was negatively affected with a decrease in mean Cq value over time when samples were transported at room temperature (Table 6). The mean Cq value and 95% CI reached in 7 days (mean value for days 1–7 taken together) were 24.00 (CI 20.90–27.10) for transportation at room temperature. However, there were no major differences between 1–3 days (difference in mean Cq value = 1.10 cycles).

The storage condition results from the study showed that the highest mean Cq values (1–30 days) were presented for samples stored at −80 °C and the lowest mean Cq values for samples stored at −20 °C. The mean Cq values in both storage conditions were stable over time. Storage in a refrigerator at +2–8 °C (1–30 days) showed a decreased analytical sensitivity with higher mean Cq values over time. For short-term storage (1–7 days), the mean Cq value and 95% CI reached in 7 days were 28.40 (CI 27.50–29.30) for storage in a refrigerator at +2–8 °C, 27.80 (CI 27.20–28.40) for storage at −20 °C, and 31.10 (CI 30.70–31.60) for storage at −80 °C. For long-term storage (1–30 days), the mean Cq value and 95% CI reached in 30 days were 29.70 (CI 28.40–31.00) for storage in refrigerator at +2–8 °C, 28.00 (CI 27.40–28.50) for storage at −20 °C, and 31.50 (CI 31.10–32.00) for storage at −80 °C. There was significant difference between storage (1–30 days) in a refrigerator at +2–8 °C and storage at −20 °C (*p* = 0.009) but also for storage in a refrigerator at +2–8 °C compared to −80 °C (*p* = 0.01), as well as for storage at −20 °C and −80 °C (*p* = <0.0001). The results showed comparable mean Cq values (<1.5 cycles in difference) for storage in a refrigerator at +2–8 °C and storage at −20 °C for storage at 1–7 days. However, these values increased over time (>7 days). The results also showed comparable mean Cq values (<1 cycle in difference) for storage in a refrigerator at +2–8 °C versus storage at −80 °C for storage of samples for 14 days or more.

#### 3.1.6. Samples with or without Leucocytosis (VI)

The results from the comparison between samples with and without elevated leucocyte count showed no major significant difference (*p* = 0.469) between the different groups (difference ranging between 0.40–1.30 cycles) (Table 7). In the samples without leucocytosis, it was possible to detect down to 10^1 cells per sample (concentration before extraction) in all samples, while in the samples with leucocytosis, detection down to 10^1 cells per sample before extraction was possible in three of four wells.

#### 3.1.7. Samples with or without Erythrocytes (VII)

The results from the comparison between samples with and without erythrocytes showed significant differences (*p* = 0.0191) between the two groups, with differences ranging between 6.80–4.10 depending on the concentration of cells in the samples (before extraction) (Table 8). In the samples with erythrocytes, it was only possible to detect down to 10^2 cells per sample before extraction in all samples, while, in the samples without erythrocytes, it was possible to detect down to 10^1 cells per sample before extraction in all samples. This indicates that erythrocytes in CSF have an inhibiting effect on the PCR reaction.

#### 3.1.8. Reproducibility and Repeatability within and between Real-Time PCR Runs (VIII)

The repeatability within one sample was high and ranged between 22.50–23.00 in Cq values (mean Cq value = 22.70) with a difference of 0.49 cycles (standard deviation = 0.17) (Table 9). In comparison between two real-time PCR analyses on the same set of samples (reproducibility), the differences ranged from 0.00–0.39 in Cq values (Table 9). The results showed that the optimised pre-analytical steps generated stable results both within and between real-time PCR analyses.

#### 3.1.9. Application of the Optimised Pre-Analytical Protocol for Different Borrelia Species (IX)

Samples consisting of pooled CSF samples spiked with three *B. burgdorferi* s.l. species (*B. afzelii* strain Lu81, *B. garinii* strain Lu59, and *B. burgdorferi* s.s. strain B31) and one *B. miyamotoi* strain (*B. miyamotoi* HT31), respectively, were analysed with the optimised protocol for the pre-analytical handling. All four strains could be detected down to concentrations of 10^1 cells per sample before extraction in all four wells (Table 10).

**Figure 1:**
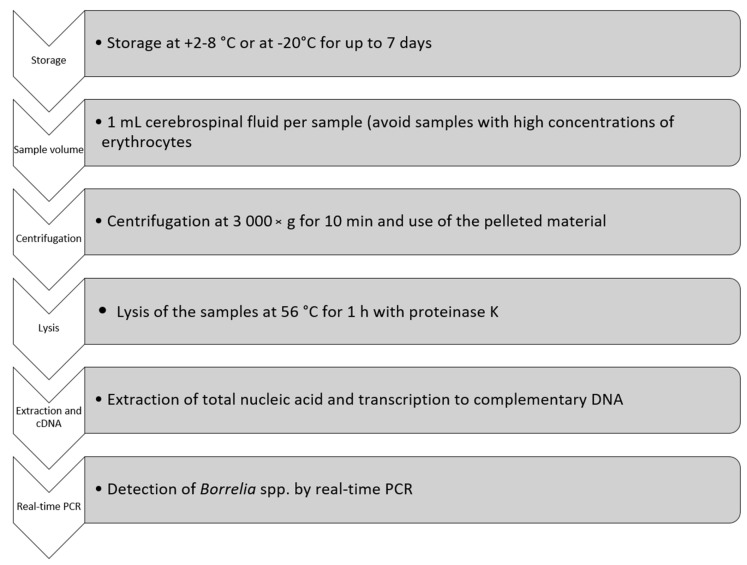
Flowchart over the optimised pre-analytical steps for detection of *Borrelia burgdorferi* sensu lato and *Borrelia miyamotoi* in cerebrospinal fluid by real-time PCR (DNA = deoxyribonucleic acid, PCR = polymerase chain reaction).

The overall results of the nine setups showed an advantage in analytical sensitivity in CSF samples, without inhibiting agents like erythrocytes, centrifuged at 3000× *g* for 10 min of which the pelleted material was used for further analysis for extraction of total NA followed by transcription to cDNA. A flowchart over the optimised pre-analytical steps for the detection of *B. burgdorferi* s. l. and *B. miyamotoi* in CSF by real-time PCR is shown in Figure 1.

## 4. Discussion

In this study, the pre-analytical procedures for subsequent detection of *B. burgdorferi* s.l. and *B. miyamotoi* in CSF by real-time PCR were systematically evaluated. The results showed that the parameters with the highest impact on the analytical sensitivity were the centrifugation time and speed, the use of cDNA as template, and the absence of erythrocytes in the sample. Based on these results, an optimised protocol for the handling of CSF samples before molecular analysis has been proposed (Figure 1).

The diagnostic sensitivity and specificity of molecular detection of *Borrelia* spirochetes in CSF are likely to depend on several factors, such as the type of template, the sample storage, the transportation conditions, the method for extraction of NA, the presence of possible PCR inhibitors, the choice of target genes, and the PCR methods. Previous studies have approached this in various ways [14,15], mainly focusing on the PCR protocols, and different pre-analytical procedures for extraction of NA and centrifugation of the samples have been used [22,23,24,25]. Therefore, the results from the different studies are difficult to compare. To our knowledge, there are no standardised recommendations regarding pre-analytical handling of CSF samples and detection methods published so far, and unsatisfactory sample preparation may be a contributing factor to the limited usefulness of PCR in routine diagnostics of LNB [14].

In most studies, the focus has been on study design (sample sizes, clinical specimens, and patient categories) and PCR methods (different target genes and primer-probe sets) to achieve high diagnostic sensitivity and specificity [22,25,26]. According to one of our previous studies [16], the analytical sensitivity and specificity among the PCR protocols currently used in Scandinavian laboratories were high. This indicates that the low diagnostic sensitivity of PCR in CSF samples, ranging from 10–30%, may have other explanations, such as a low amount of *Borrelia* spirochetes in the CSF, or the use of sub-optimal pre-analytic sample preparation procedures [2,26].

One of the central parameters to increase the analytical sensitivity, according to our study, is to extract NA from the pelleted material obtained after centrifugation, compared to NA extraction from the supernatant. The results from our study show that the use of pelleted material has a high impact on the analytical sensitivity (Table 2). In previous studies [22,24,25,26], the pelleted materials have been used for extraction, but no comparison in diagnostic or analytic sensitivity between pelleted material and the supernatant has been reported. However, even though no clinical evaluation was performed in this study we propose the use of pelleted material for detection of *Borrelia* bacteria in CSF samples based on the raised analytical sensitivity in this study.

In order to separate the pelleted material from the supernatant, centrifugation is needed. The results from our study showed that a more moderate centrifugation time and speed yielded an analytical sensitivity comparable to previous studies using a high centrifugation speed and a long centrifugation time [22,26] (Table 3). A shorter centrifugation time may actually be preferable, especially in clinical practice. The lowest analytical sensitivity was shown after using maximal centrifugation speed for one hour. However, this may also be the result of a raised temperature obtained in the centrifuge (if not using a centrifuge with the possibility of temperature control), which may have a negative influence on the NA. In the study presented here, there was no comparison between non-centrifuged spiked CSF and CSF samples centrifuged at different conditions. However, a previous pilot study was performed showing advantage in using the pelleted material for extraction compared to the supernatant (data not shown). The results from the pilot study showed the Cq values between non-centrifuged CSF and CSF centrifuged at 500× *g* for 5 min were comparable, while there was a major difference between centrifuged samples and non-centrifuged CSF when the time and speed was increased. Based on these results, we chose to focus on comparing different centrifugation conditions in the present study.

In our study, using primers and probes targeting *16S* rRNA, extraction of total NA followed by transcription to cDNA generated a higher analytical sensitivity with lower mean Cq values compared to the use of DNA (Table 4). Theoretically, extraction of total NA followed by transcription to cDNA may result in a higher number of target DNA molecules per cell, since each sample will contain a mix of extracted DNA and cDNA [27]. However, the increased sensitivity for samples extracted for total NA depends on the use of primers aimed at target gene *16S* rRNA according to a study by Lager et al. (2017) [16]. Additionally, one must be aware that the use of cDNA as template is more time-consuming, expensive, and laborious, which may be a disadvantage for clinical laboratories since this may lead to extended turnaround time. However, we consider these disadvantages as minor compared to the increased chances of detecting *Borrelia* bacteria in CSF and thereby proving important information for correct diagnosis and treatment in cases where serology in negative. In previous studies, lysis with proteinase K and extraction of DNA has been used, while extraction of total NA is more uncommon [22,24,25,26]. However, no evaluation regarding the analytical performance of the different methods has been made so far [28].

Furthermore, our study showed that the difference in mean Cq values between the different sample volumes (0.3–0.5 mL, 0.5–1.0 mL, and 1.0–2.0 mL) was low (Table 5). In our protocol, a sample volume of 1.0 mL was proposed. However, since there were no major differences between 0.5 and 1.0 mL, both volumes are suitable to use, even though 0.5 mL may be preferable in clinical practice, especially in paediatric patients from whom smaller sample volumes are often obtained. However, the use of less than 0.5 is not recommended from our experience. Previous studies have used different sample volumes [22,24,25,26], but no recommendations have been presented so far. In a study by Forselv et al. (2018) [23], an effort has been made to evaluate the influence of different CSF volumes. However, the results from the study showed no increased diagnostic sensitivity using a larger volume of sample, which is in line with the results in our study. In addition, the available sample volumes are generally limited and are often intended to be used for several analyses. Therefore, the use of a smaller CSF volume (0.5 mL) would be preferable.

The results from our study showed a decreased analytical sensitivity in the PCR assay for short-termed storage (1–7 days) and transportation of samples at room temperature (Table 6). However, the differences in Cq values between 1 and 3 days were low, indicating that samples can be transported at room temperature if the samples are stored in a refrigerator before transportation and immediately upon arrival at the laboratory. When the samples have arrived at the laboratory, short-term storage can be done safely at +2–8 °C up to at least 3 days according to the present study. These findings are in line with previous studies [17,29] but also with instructions from several manufacturers of commercial kits, showing that *Borrelia* cells are stable without any significant decrease in Cq value at +4 °C for 0–7 days. In a study by Forslev et al. (2018) [23], the centrifugation and the freezing of the pelleted material was performed within 24 h, however with no raised diagnostic sensitivity reported in adult patients with LNB compared to previous presented results [2].

For long-term storage, the results from our study indicate that storage at −20 °C should be recommended (Table 6). However, the results from a previous study [30], but also from some manufacturers of commercial PCR kits regarding storage at −80 °C, differ from the results found in our study, demonstrating significantly lower analytical PCR sensitivity in samples stored at −80 °C compared to −20 °C. This may be the result of degradation of mRNA molecules since storage at −80 °C does not keep the mRNA intact. Similar results were also found in a study by Huang et al. (2017) [31]. Storage at temperatures lower than −20 °C can, apart from degradation of mRNA, also cause damage to the fragile spirochetes [30]. In order to fully evaluate the influence of freezing temperature on subsequent PCR sensitivity related to degradation of mRNA, samples stored at low temperature (<−20 °C) followed by extraction of both DNA and total NA should be compared. Based on results from that comparison, conclusions can be drawn regarding temperature for long-term storage in relation to the type of template.

The outcome of our study shows that samples with a large number of erythrocytes generated lower analytical sensitivity in the PCR analysis (Table 8). This result is in line with the findings in a previous study by Al-Soud et al. 2001 [32], suggesting that several compounds in the blood, including haemoglobin and lactoferrin, in erythrocytes and in leucocytes, respectively, may serve as major PCR inhibitors. The risk of false negative PCR results is something to be aware of when analysing and reporting results obtained from PCR analysis of CSF samples with visible signs of erythrocytes. In our study, erythrocyte concentration was estimated based on visual colour change, and the critical number of erythrocytes in a CSF sample that causes inhibition has not been identified. It would be interesting to further evaluate at which erythrocyte concentration the analytical sensitivity is decreased.

Physiological properties of *Borrelia* spirochetes may differ between cultured bacteria and bacteria during mammal infection, which is a limitation in our study. We believe however, that our approach of using CSF samples spiked with a known amount of culture *Borrelia* bacteria is the most suitable approach to investigate how the analytical sensitivity and specificity of the PCR methods is influenced by different pre-analytical parameters. Another way to address the issue is to use clinical samples from patients diagnosed with LNB. Such an approach, however, is more uncertain since the number of *Borrelia* spirochetes is unknown, making it difficult to evaluate the influence of the different parameters, especially in samples with a low number of *Borrelia* bacteria. It is also challenging to acquire the large amount of CSF required for testing all parameters from the same patient since the sample material is primarily used for routine diagnosis of LNB but also for diseases not correlated to LNB. Another limitation in our study is the use of more than one bacterial culture due to the large number of setups. However, within each setup, the same bacterial culture was used and counted in duplicates in each culture by the same person during our entire study, thus allowing a reliable comparison of specific parameters within that setup.

PCR is not recommended to be used as a primary laboratory method for diagnosis of LNB, and our study was not intended to change that recommendation. However, in some specified cases, PCR can be used as a complement to the conventional serology, like in early cases of acute LNB, when the antibodies have not yet been developed and serologic tests therefore give false negative results. Earlier studies on LNB have shown a tendency of more positive PCR results from patients with a short duration of symptoms (<14 days), and PCR may in these cases serve as a diagnostic supplement to serology [33]. PCR can also be used to confirm LNB in patients with intrathecal *Borrelia*-specific antibodies remaining from a previous episode of LNB, since these antibodies, which are known to persist for years after the infection [34], may complicate interpretation of serological results. As a contrast, PCR is the primary clinical diagnostic method for detection of *B. miyamotoi* in CSF, as well as in serum or plasma, since no commercial serological tests are currently available [7].

In this study, a systematic evaluation of the pre-analytical conditions was performed with the aim of increasing the analytical sensitivity of the subsequent PCR analysis for detection of *Borrelia* spirochetes in CSF samples, and an optimised protocol for pre-analytical handling was established. Our study suggests that the most important steps for increased analytical sensitivity are the concentration step (centrifugation) and the use of the pelleted material, the use of cDNA as template, and the absence of erythrocytes in the CSF samples. The new optimised pre-analytical protocol (Figure 1) indicated an improved analytical sensitivity. However, further evaluation on well-characterised clinical samples from patients with LNB, but also in patients with *B. miyamotoi* meningoencephalitis, is recommended to improve the clinical diagnostics.

## Figures and Tables

**Table 1:** Conditions for each experimental setup with purpose to investigate the pre-analytical handling of cerebrospinal fluid samples from patients with suspected Lyme neuroborreliosis in order to increase the diagnostic sensitivity.

Experimental Setup	Parameter	Species	Extraction (D or S)	Concentration Range (Cells Per Sample before Extraction)	Centrifugation Material (P vs. SU)	Centrifugation Time (min)	Centrifugation Speed (˟ g)	Extraction Kit ^1^	Template Type (cDNA and DNA)	Sample Volume (mL)	Storage Temperature (°C) ^2^	Storage (Days)	Leucocytes (L vs. NL)	Erythrocytes (E vs. NE)
I	Concentration	*B. garinii*	D	10^4−10^0	P and SU	10	3000	**Total NA**	cDNA	1.0	No storage	0	NL	NE
II	Centrifugation (time and speed)	*B. garinii*	D	10^4−10^0	P	(1) 60 (2) 10 (3) 60 (4) 5 (5) non centrifuged	(1) 10,000 (2) 3000 (3) 18,840 (4) 500 (5) -	**Total NA**	cDNA	1.0	No storage	0	NL	NE
III	Type of template	*B. garinii*	D	10^4−10^0	P	10	3000	**Total NA** and DNA	cDNA and DNA	1.0	No storage	0	NL	NE
IV	Sample volume	*B. garinii*	D	10^3	P	10	3000	**Total NA**	cDNA	(1) 0.3 (2) 0.5 (3) 1.0 (4) 2.0	No storage	0	NL	NE
V	Storage and transportation *	*B. garinii*	D	10^3	P	10	3000	**Total NA**	cDNA	1.0	(1) No storage (ST) (2) Room temperature (T) (3) Refrigerator (2–8 °C) (ST) (4) Freezer (−20 °C) (ST) (5) Low-temperature freezer (−80 °C) (ST)	0 1 3 7 14 30	NL	NE
VI	Leucocytes	*B. garinii*	D	10^4−10^0	P	10	3000	**Total NA**	cDNA	1.0	No storage	0	L and NL	NE
VII	Erythrocytes	*B. garinii*	D	10^4−10^0	P	10	3000	**Total NA**	cDNA	1.0	No storage	0	NL	E and NE
VIII	Reproducibility /repeatability	*B. garinii*	S	10^4−10^0	P	10	3000	**Total NA**	cDNA	1.0	No storage	0	NL	NE
IX	Different strains	*B. garinii* *B. afzelii* *B. burgdorferi sensu stricto* *B. miyamotoi*	D	10^4−10^0	P	10	3000	**Total NA**	cDNA	1.0	No storage	0	NL	NE

D = duplicates, S = single, P = pelleted material, SU = supernatant, NA = nucleic acid, DNA = deoxyribonucleic acid, cDNA = complementary DNA, L = leucocytes, NL = non-leucocytes, E = erythrocytes, NE = non erythrocytes, B = *Borrelia*, T= transportation, ST = storage. ^1^. EZ1 RNA Tissue Mini Kit (total NA) and EZ1 DNA Tissue Mini Kit (DNA) (Qiagen, Hilden, Germany). ^2^. No storage = the cerebrospinal fluid was thawed and spiked with diluted cultured bacteria, and no further storage was performed. * Storage and transportation conditions were evaluated at separate occasions with different sample setups.

**Table 2:** Comparison of the analytical sensitivity between extraction of nucleic acid from the supernatant versus extraction of nucleic acid from the pelleted material.

Concentration (Cells Per Sample before Extraction)	Supernatant, Mean Cq Value, (SD) *	Pelleted Material, Mean Cq Value, (SD) *	Difference in Mean Cq Value, (Cq^supernatant^ − Cq ^pelleted material^)
10^4	30.30 (0.70)	22.30 (0.81)	8.00
10^3	34.20 (1.70)	26.00 (0.96)	8.20
10^2	-	29.80 (0.81)	-
10^1	-	37.20 ^#^ (NC)	-
10^0	-	-	-

NC = not calculated. SD = standard deviation. Cq = quantification cycle value. # = one of four samples amplified. * = Mean Cq values and standard deviations are based on two extractions where each eluate is placed in two wells each per real-time PCR resulting in four wells per dilution.

**Table 3:** Comparison of four different centrifugation conditions (time and speed).

Concentration (Cells Per Sample before Extraction)	Centrifugation Time (min)	Centrifugation Speed (× *g*) ^Ω^	Mean Cq Value, (SD) *
10^4	60	10,000	22.10 (0.75)
10^3	60	10,000	26.20 (0.52)
10^2	60	10,000	31.60 (0.64)
10^1	60	10,000	35.40 (0.51)
10^0	60	10,000	34.10 ^#^ (NC)
			
10^4	10	3000	22.60 (0.49)
10^3	10	3000	26.50 (0.48)
10^2	10	3000	31.60 (0.62)
10^1	10	3000	34.90 (1.20)
10^0	10	3000	-
			
10^4	60	18,840	24.90 (0.31)
10^3	60	18,840	27.60 (0.20)
10^2	60	18,840	33.80 (0.37)
10^1	60	18,840	37.20 ^&^ (NC)
10^0	60	18,840	-
			
10^4	5	500	22.50 (0.45)
10^3	5	500	25.80 (0.38)
10^2	5	500	31.80 (0.36)
10^1	5	500	37.20 ^#^ (NC)
10^0	5	500	-

Ω = maximal speed for the centrifuge was 18,840× *g*. NC = not calculated. SD = standard deviation. Cq = quantification cycle value. # = one of four samples amplified. & = two of four samples amplified. * = Mean Cq values and standard deviations are based on two extractions where each eluate is placed in two wells each per real-time PCR resulting in four wells per dilution.

**Table 4:** Comparison between the uses of DNA versus complementary DNA as template.

Concentration (Cells Per Sample before Extraction)	EZ1 RNA Tissue Mini Kit, Mean Cq Value, (SD) *	EZ1 DNA TISSUE Mini Kit), Mean Cq Value, (SD) *	Difference in Mean Cq Value (Cq^RNA^ − Cq^DNA^)
10^4	22.00 (0.45)	24.10 (0.11)	2.10
10^3	25.60 (0.21)	28.70 (0.81)	3.10
10^2	29.80 (0.46)	30.80 (0.55)	1.00
10^1	35.10 (0.29)	36.90 (0.65)	1.80
10^0	-	38.10 ^#^ (NC)	-

NC = not calculated. SD = standard deviation. Cq = quantification cycle value. # = one of four samples amplified. * = Mean Cq values and standard deviations are based on two extractions where each eluate is placed in two wells each per real-time PCR resulting in four wells per dilution.

**Table 5:** Comparison of four different sample volumes for detection of *Borrelia burgdorferi* sensu lato in cerebrospinal fluid.

	Difference in Mean Cq Values				Mean Cq Value, (SD) *
**Volume (mL)**	**0.3**	**0.5**	**1.0**	**2.0**	
**0.3**		0.90	1.90	2.50	28.30 (0.09)
**0.5**	0.90		1.00	1.60	27.40 (0.41)
**1.0**	1.90	1.00		0.60	26.40 (0.14)
**2.0**	2.50	1.60	0.60		25.80 (0.14)

SD = standard deviation. Cq = quantification cycle value. * = mean Cq values, and standard deviations are based on two extractions where each eluate is placed in two wells each per real-time PCR resulting in four wells per dilution.

**Table 6:** Comparison between different storage and transport conditions for 0–30 days at four different temperatures.

Storage and Transportation Conditions (Temperature)	Storage/Transportation (ST Versus T)	Day 0, Mean Cq Value, (SD) *	Day 1, Mean Cq Value, (SD) *	Day 3, Mean Cq Value, (SD) *	Day 7, Mean Cq Value, (SD) *	Day 14, Mean Cq Value, (SD) *	Day 30, Mean Cq Value, (SD) *
No storage **		27.30 (0.15)	NP	NP	NP	NP	NP
Room temperature **	T	NP	22.80 (0.06)	23.90 (0.06)	25.30 (0.12)	NP	NP
Refrigerator (+2–8 °C)	ST	NP	27.70 (0.26)	28.20 (0.17)	29.50 (0.19)	31.60 (0.82)	31.60 (0.23)
Freezer (−20 °C)	ST	NP	27.90 (0.41)	27.30 (0.17)	28.10 (0.56)	29.20 (0.14)	27.50 (0.06)
Low-temperature freezer (−80 °C)	ST	NP	31.10 (0.30)	30.80 (0.30)	31.50 (0.18)	32.20 (0.61)	32.10 (0.31)

ST = storage. T = transportation. NP = not presented. SD = standard deviation. Cq = quantification cycle value. * = mean Cq values and standard deviations are based on two extractions where each eluate is placed in two wells each per real-time PCR resulting in four wells per dilution. ** = Mean value/values are based on one extraction where the eluate is placed in two wells each per real-time PCR.

**Table 7:** The difference in mean quantification cycle values in cerebrospinal fluid samples with leucocytosis versus without leucocytosis.

Concentration (Cells Per Sample before Extraction)	Leucocytosis ^¤^, Mean Cq Value, (SD) *	Without Leucocytosis, Mean Cq Value, (SD) *	Difference in Mean Cq Values (Cq^leucocytosis^ − Cq^non leucocytosis^)
10^4	22.50 (0.90)	21.30 (0.70)	1.20
10^3	25.30 (0.32)	24.00 (0.36)	1.30
10^2	28.60 (0.38)	28.20 (0.30)	0.40
10^1	34.00 (1.72) ^¶^	35.10 (1.45)	1.10
10^0	-	-	-

SD = standard deviation. Cq = quantification cycle value. ¤ = mononuclear cell count (leucocytosis) >5 × 10^6/L. ¶ = three of four samples amplified. * = mean Cq values and standard deviations are based on two extractions where each eluate is placed in two wells each per real-time PCR resulting in four wells per dilution.

**Table 8:** The difference in mean quantification cycle values between cerebrospinal fluid sample with and without erythrocytes.

Concentration (Cells Per Sample before Extraction)	Erythrocytes ^$^, Mean Cq Value, (SD)*	Without Erythrocytes, Mean Cq Value, (SD) *	Difference in Mean Cq Values (Cq^erythrocytes^ − Cq^non erythrocytes^)
10^4	28.10 (0.79)	21.30 (0.70)	6.80
10^3	29.30 (0.22)	24.00 (0.36)	5.30
10^2	32.40 (0.23)	28.30 (0.30)	4.10
10^1	-	35.10 (1.45)	-
10^0	-	-	-

SD = standard deviation. Cq = quantification cycle value. $ = mononuclear cell count (erythrocytes) >5 × 10^6/L. * = Mean Cq values and standard deviations are based on two extractions where each eluate is placed in two wells each per real-time PCR resulting in four wells per dilution.

**Table 9:** Repeatability for one sample and reproducibility between two real-time PCR runs on the same set of samples.

Sample ID	Between or within Analyses (B/W)	Mean Cq Value, (SD) ^α^ (Analysis 1)	Mean Cq Value, (Analysis 2)	Difference in Mean Cq (Cq^analysis 1^−Cq^analysis 2^)
1	B	22.80	23.20	0.39
2	B	22.30	22.60	0.28
2	W	22.70 (0.17)		
2	W			
2	W			
2	W			
2	W			
2	W			
2	W			
2	W			
3	B	22.80	23.00	0.21
4	B	25.30	25.50	0.21
5	B	23.90	24.00	0.09
6	B	22.70	22.70	0.03
7	B	23.00	22.70	0.17
8	B	26.10	26.00	0.08
9	B	25.30	25.20	0.09
10	B	22.70	22.60	0.07
11	B	22.60	22.60	0.00
12	B	26.20	26.20	0.00

B = between. W = within. SD = standard deviation. Cq = quantification cycle value. ^α^ = mean value and standard deviation are only calculated for the comparison within one sample.

**Table 10:** Optimised protocol for pre-analytical handling applied to three *Borrelia burgdorferi* sensu lato strains (*Borrelia afzelii* Lu81, *Borrelia garinii* Lu59, and *B. burgdorferi* sensu stricto B31) and one *Borrelia miyamotoi* strain (*B. miyamotoi* HT31).

Concentration (Cells Per Sample before Extraction)	*B. garinii* Lu59, Mean Cq Value, (SD) *	*B. burgdorferi* Sensu Stricto B31, Mean Cq Value, (SD) *	*B. afzelii* Lu81, Mean Cq Value, (SD) *	*B. miyamotoi* HT31, Mean Cq Value, (SD) *
10^4	22.60 (0.49)	20.40 (0.27)	21.40 (0.11)	23.00 (0.53)
10^3	26.50 (0.48)	23.90 (0.20)	26.40 (0.18)	26.20 (1.16)
10^2	31.60 (0.62)	28.00 (0.17)	29.30 (0.11)	29.50 (0.58)
10^1	34.90 (1.20)	35.30 (0.46)	33.90 (1.39)	34.50 (0.25)
10^0	-	-	-	-

SD = standard deviation. Cq = quantification cycle value. B. = *Borrelia*. * = mean Cq values and standard deviations are based on two extractions where each eluate is placed in two wells each per real-time PCR resulting in four wells per dilution.

## Supplementary Materials

The following are available online at https://www.mdpi.com/article/10.3390/diagnostics11112088/s1, Supplementary Materials provides more detailed information regarding each setup presented in the materials and methods section.
