# Supplementary material for: Molecular Detection of Borrelia Bacteria in Cerebrospinal Fluid-Optimisation of Pre-Analytical Sample Handling for Increased Analytical Sensitivity
Source: Diagnostics (Basel). 2021 Nov 12;11(11):2088. doi: 10.3390/diagnostics11112088 (PMC8623531; doi:10.3390/diagnostics11112088)
Supplement: Supplementary file 1 [file diagnostics-11-02088-s001.zip › diagnostics-1433386-supplementary.pdf]

Detailed information regarding setup I-VII

Detailed information regarding each setup are described in the text below, while a more simplified version is presented in Table 1 in the paper.

Concentration (use of supernatant and pelleted material)

- Initially a dilution series ranging from 2 × 10^-1 to 2 × 10^3 cells μL^{-1} for *B. garinii* strain Lu 59 was prepared. By applying 5 μL per dilution to 1 mL cerebrospinal fluid (CSF) (without leucocytosis or erythrocytes) a final concentration of 10^0 to 10^4 cells per sample before extraction was established. Each concentration was extracted in duplicates.
- The samples were evaluated as fresh samples *i.e.* without storage after bacterial spiking.
- Each sample was centrifuged at 3000 x g for 10 minutes.
- A total of 300 μL supernatant was applied to a 2.0 mL sample vial for further extraction. Of the remaining sample, 400 μL was discarded, while the remaining 300 μL supernatant including the pelleted material was used for further extraction.
- Both supernatant and supernatant including pelleted material was extracted as duplicates for each dilution.
- Each sample was lysed by adding 20 μL proteinase K (Qiagen, Hilden, Germany) to the vials followed by mix on vortex (Vortex-Genie®2, Scientific Industries, Inc., Bohemia, NY), and lysis at 56 °C for 1 hour.

- The samples was extracted for total nucleic acid (NA) using the EZ1 RNA Tissue Mini Kit (Qiagen) according to manufacturer´s instructions. All extractions were performed on the EZ1 Advanced XL instrument (Qiagen), with an elution volume of 50 µL.
- After extraction, 15 µL of the total NA were reverse-transcribed to complementary deoxyribonucleic acid (cDNA) by using Illustra™ Ready-to-Go RT-PCR beads kit (GE Healthcare Life Science, Amersham, Place, UK) as previously described by Lager et al. (2017) [16]. The cDNA synthesis was prepared as single samples.
- A flowchart of the entire process is presented in Figure S1.

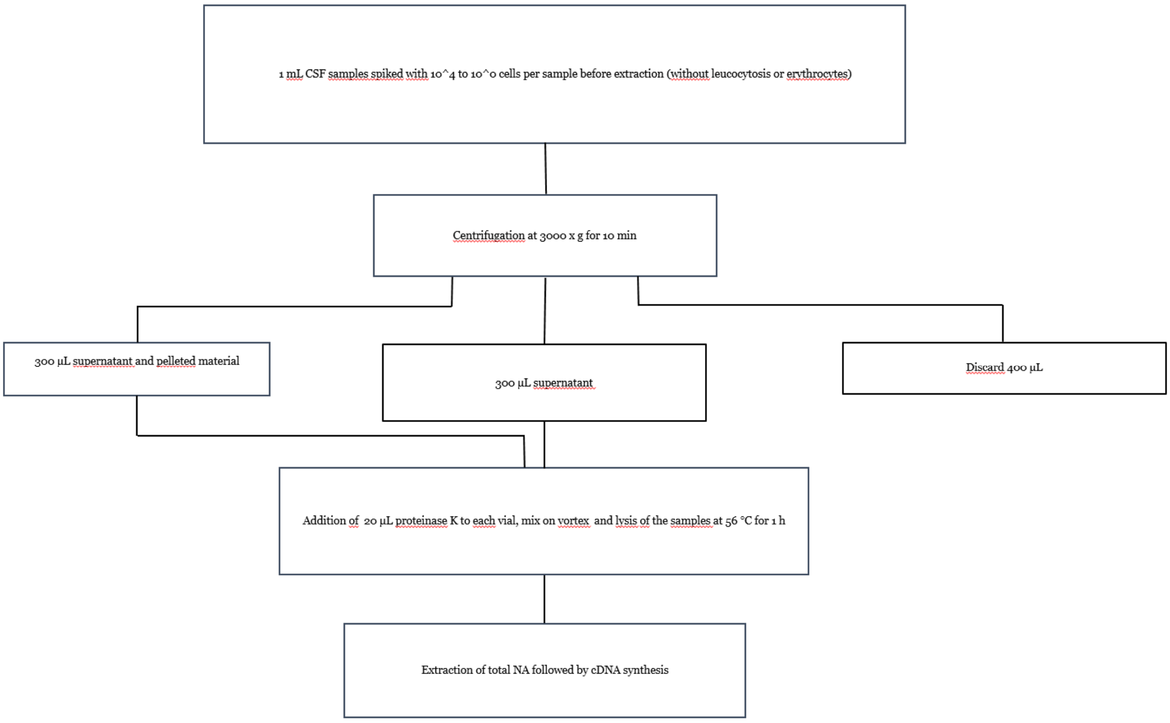

Figure S1: Flowchart for setup I evaluating concentration (use of supernatant and pelleted material) (CSF=cerebrospinal fluid, NA=nucleic acid, cDNA= complementary deoxyribonucleic acid)

Centrifugation (time and speed)

- Initially a dilution series ranging from 2 × 10^-1 to 2 × 10^3 cells μL^{-1} for *B. garinii* strain Lu 59 was prepared. By applying 5 μL per dilution to 1 mL CSF (without leucocytosis or erythrocytes) a final concentration of 10^0 to 10^4 cells per sample before extraction was established.
- The samples were evaluated as fresh samples *i.e.* without storage after bacterial spiking.
- Each dilution, extracted as duplicated, was evaluated for four different centrifugation conditions. For more information, see Table 1.
- After centrifugation, 700 μL supernatant was discarded. The remaining 300 μL supernatant including the pelleted material was lysed by adding 20 μL proteinase K (Qiagen) to the vials followed by mix on vortex (Scientific Industries, Inc.), and lysis at 56 °C for 1 hour.
- Each sample was extracted for total NA using the EZ1 RNA Tissue Mini Kit (Qiagen) according to manufacturer´s instructions. All extractions were performed on the EZ1 Advanced XL instrument (Qiagen), and the elution volume was 50 μL.
- After extraction, 15 uL of the total NA were reverse-transcribed to cDNA by using Illustra™ Ready-to-Go RT-PCR beads kit (GE Healthcare Life Science, Amersham, Place, UK) as previously described by Lager *et al.* (2017) [16]. The cDNA synthesis was prepared as single samples.
- A flowchart of the entire process is presented in Figure S2.

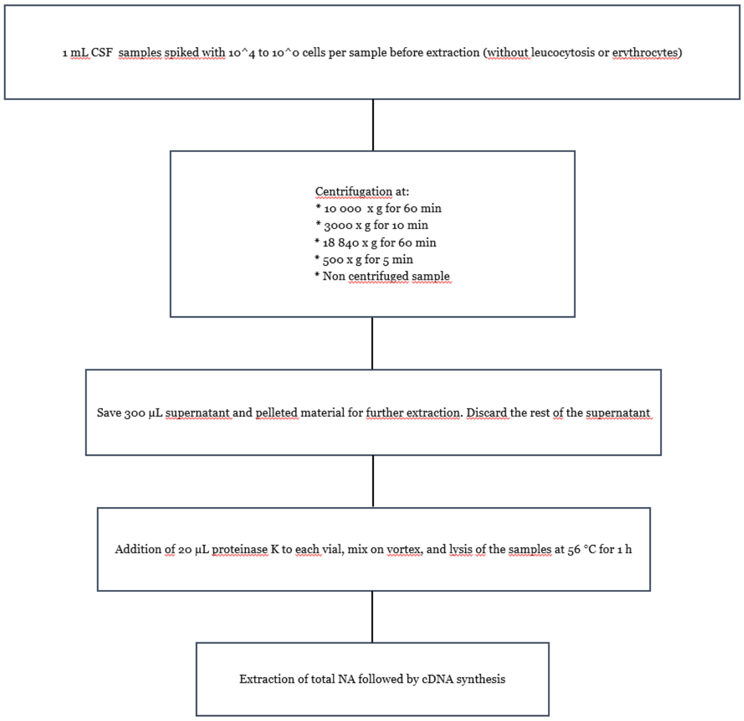

Figure S2: Flowchart for setup II evaluating centrifugation (time and speed)

(CSF=cerebrospinal fluid, NA=nucleic acid, cDNA= complementary deoxyribonucleic acid)

Extraction methods and template type

- Initially a dilution series ranging from 2 × 10^-1 to 2 × 10^3 cells μL^{-1} for *B. garinii* strain Lu 59 was prepared. By applying 5 µL per dilution to 1 mL CSF (without leucocytosis or erythrocytes) a final concentration of 10^0 to 10^4 cells per sample before extraction was established. Each sample was extracted in duplicates for each extraction method.
- The samples were evaluated as fresh samples *i.e.* without storage after bacterial spiking.
- All samples were centrifuged at 3000 x g for 10 minutes.

- For the extraction of total NA, 700 µL supernatant was discarded. The remaining 300 µL supernatant including the pelleted material was used for further extraction.
- For the extraction of DNA, 800 µL supernatant was discarded. The remaining 200 µL supernatant including the pelleted material was used for further extraction.
- Two vials per dilution was extracted for total NA and two for DNA.
- To each vial, 20 µL proteinase K (Qiagen) was added followed by mix by vortex (Scientific Industries, Inc.), and lysis at 56 °C for 1 hour.
- The samples extracted for total NA used the EZ1 RNA Tissue Mini Kit (Qiagen) and the samples extracted for DNA used the EZ1 DNA Tissue Mini Kit (Qiagen), both according to manufacturer´s instructions.
- All extractions were performed on the EZ1 Advanced XL instrument (Qiagen), with an elution volume of 50 µL.
- After extraction, 15 uL of the total NA were reverse-transcribed to cDNA by using Illustra™ Ready-to-Go RT-PCR beads kit (GE Healthcare Life Science) as previously described by Lager *et al.* (2017) [16].
- For each sample extracted for total NA, three cDNA syntheses reactions were performed for the entire amount of eluate (total NA). The cDNA samples generated were pooled to create a homogenous sample with 100 % yield of the entire elution, which was compared to the extraction of DNA.

- A flowchart of the entire process is presented in Figure S3.

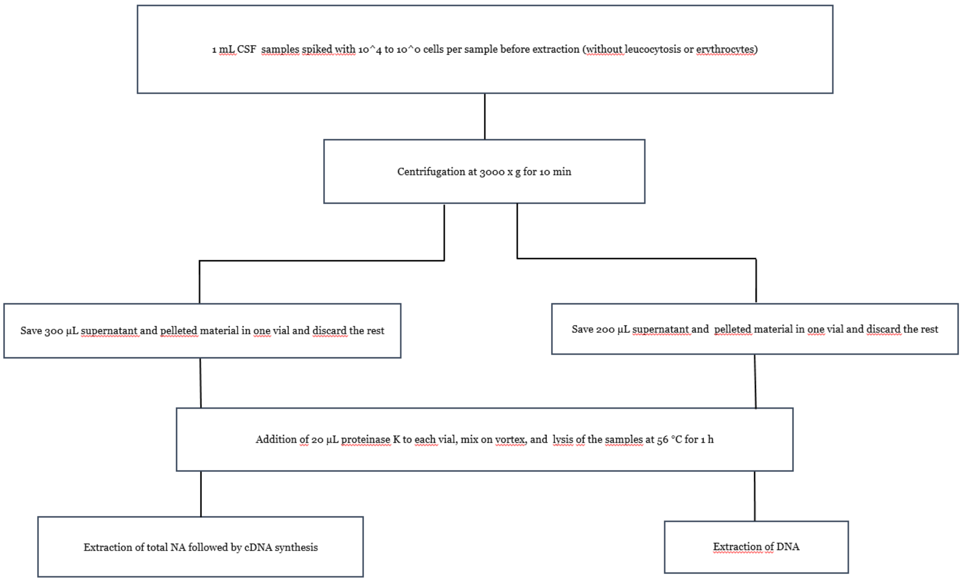

Figure S3: Flowchart for setup III evaluating extraction methods and template type (CSF=cerebrospinal fluid, NA=nucleic acid, DNA= deoxyribonucleic acid, cDNA= complementary DNA)

Sample volume

- Initially a dilution series ranging from 2 × 10^-1 to 2 × 10^3 cells µL^{-1} for *B. garinii* strain Lu 59 was prepared. By applying 5 µL of dilution 2 × 10^2 cells µL^{-1} to 0.3, 0.5, 1.0, or 2.0 mL CSF samples (without leucocytosis or erythrocytes) a final concentration of 10^3 cells per sample before extraction was established. Each volume was extracted in duplicates.
- The samples were evaluated as fresh samples *i.e.* without storage after bacterial spiking.
- All sample was prior to extraction centrifuged at 3000 x g for 10 minutes.

- In total, 300 µL of the supernatant including the pelleted material was saved for further extraction while the rest was discarded. The samples containing 0.3 mL was centrifuged, even though it was not necessary for concentration of the sample, to ensure the same treatment for all four sample volumes.
- Each sample was lysed by adding 20 µL proteinase K (Qiagen) to the vials followed by mix by vortex (Scientific Industries, Inc.), and lysis at 56 °C for 1 hour.
- The samples were extracted for total NA using the EZ1 RNA Tissue Mini Kit (Qiagen) according to manufacturer´s instructions. All extractions were performed on the EZ1 Advanced XL instrument (Qiagen), with an elution volume of 50 µL.
- After extraction, 15 uL of the total NA were reverse-transcribed to cDNA by using Illustra™ Ready-to-Go RT-PCR beads kit (GE Healthcare Life Science, Amersham, Place, UK) as previously described by Lager *et al.* (2017) [16]. The cDNA synthesis was prepared as single samples.
- A flowchart of the entire process is presented in Figure S4.

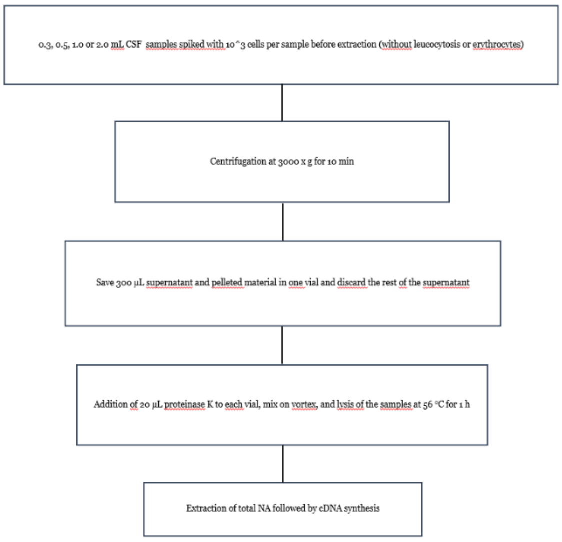

Figure S4: Flowchart for setup IV evaluating the sample volume (CSF=cerebrospinal fluid, NA=nucleic acid, cDNA= complementary deoxyribonucleic acid)

Storage and transportation

- Initially a dilution series ranging from 2 ˟ 10^-1 to 2 ˟ 10^3 cells μL^{-1} for *B. garinii* strain Lu 59 was prepared. By applying 5 μL of dilution 2 ˟ 10^2 cells μL^{-1} to 1 mL CSF (without leucocytosis or erythrocytes) a final concentration of 10^3 cells per sample before extraction was established. Each storage and transportation condition were extracted as duplicates.
- The storage conditions were evaluated in six different sets of time (0, 1, 3, 7, 14, and 30 days) and three different temperatures (2-8 °C, -20 °C, and -80 °C), while the transportation conditions were evaluated at four sets of time (0, 1, 3, and 7 days) and one temperature (room temperature).
- All sample was prior to extraction centrifuged at 3000 x g for 10 minutes.
- A total of 700 μL supernatant was discarded and the remaining sample, 300 μL supernatant including the pelleted material, was used for further extraction.
- Each sample was lysed by adding 20 μL proteinase K (Qiagen) to the vials followed by mix by vortex (Scientific Industries, Inc.), and lysis at 56 °C for 1 hour.
- The samples were extracted for total NA using the EZ1 RNA Tissue Mini Kit (Qiagen) according to manufacturer´s instructions. All extractions were performed on the EZ1 Advanced XL instrument (Qiagen), with an elution volume of 50 μL.

- After extraction, 15 uL of the total NA were reverse-transcribed to cDNA by using Illustra™ Ready-to-Go RT-PCR beads kit (GE Healthcare Life Science, Amersham, Place, UK) as previously described by Lager *et al.* (2017) [16]. The cDNA synthesis was prepared as single samples.
- A flowchart of the entire process is presented in Figure S5.

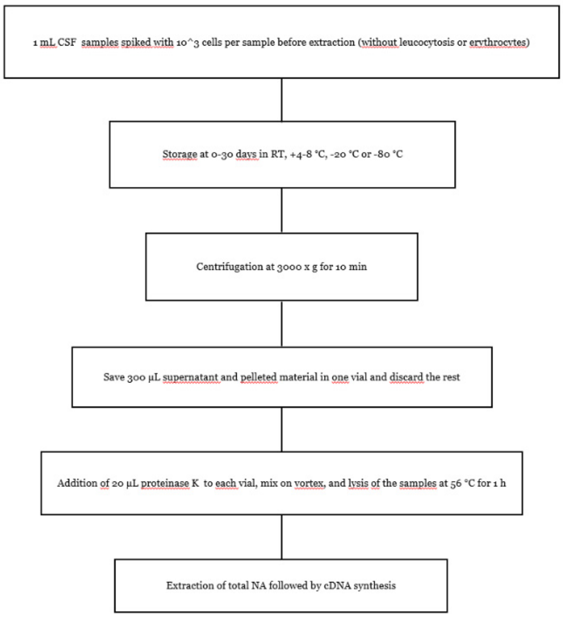

Figure S5: Flowchart for setup V evaluating storage and transportation (RT=room temperature, CSF=cerebrospinal fluid, NA=nucleic acid, cDNA= complementary deoxyribonucleic acid)

Leucocytosis

- Initially a dilution series ranging from 2 × 10^-1 to 2 × 10^3 cells μL^{-1} for *B. garinii* strain Lu 59 was prepared. By applying 5 µL per dilution to 1 mL CSF sample with leucocytosis and 1 mL CSF sample without leucocytosis (both without

erythrocytes) a final concentration of 10^0 to 10^4 cells per sample before extraction was established. Each dilution was extracted in duplicates.

- The samples were evaluated as fresh samples *i.e.* without storage after bacterial spiking.
- All sample was prior to extraction centrifuged at 3000 x g for 10 minutes.
- A total of 700 μL supernatant was discarded and the remaining sample, 300 μL supernatant including the pelleted material, was used for further extraction.
- Each sample was lysed by adding 20 μL proteinase K (Qiagen) to the vials followed by mix by vortex (Scientific Industries, Inc.), and lysis at 56 °C for 1 hour.
- The samples were extracted for total NA using the EZ1 RNA Tissue Mini Kit (Qiagen) according to manufacturer´s instructions. All extractions were performed on the EZ1 Advanced XL instrument (Qiagen), with an elution volume of 50 μL.
- After extraction, 15 uL of the total NA were reverse-transcribed to cDNA by using Illustra™ Ready-to-Go RT-PCR beads kit (GE Healthcare Life Science, Amersham, Place, UK) as previously described by Lager *et al.* (2017) [16]. The cDNA synthesis was prepared as single samples.

- A flowchart of the entire process is presented in Figure S6.

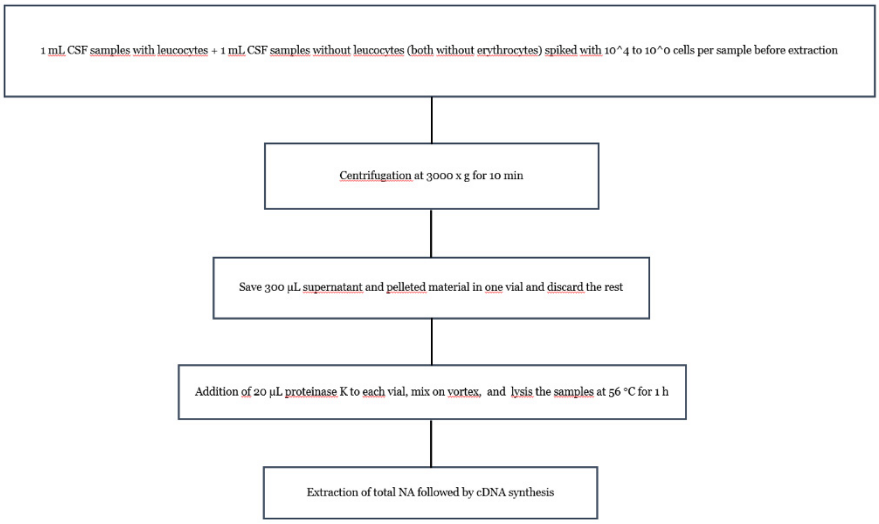

Figure S6: Flowchart for setup VI evaluating the influence of leucocytosis (CSF=cerebrospinal fluid, NA=nucleic acid, cDNA= complementary deoxyribonucleic acid)

Erythrocytes

- Initially a dilution series ranging from 2 ˣ 10^-1 to 2 ˣ 10^3 cells µL^{-1} for *B. garinii* strain Lu 59 was prepared. By applying 5 µL per dilution to 1 mL CSF with erythrocytes and 1 mL CSF sample without erythrocytes (both without leucocytosis) a final concentration of 10^0 to 10^4 cells per sample before extraction was established. Each dilution was extracted as duplicates.
- To create CSF samples with erythrocytes, 2.5 µL ethylenediaminetetraacetic acid blood was added to half of the samples, resulting in a pink colour.
- The samples were evaluated as fresh samples *i.e.* without storage after bacterial spiking.
- All sample was prior to extraction centrifuged at 3000 x g for 10 minutes.

- A total of 700 µL supernatant was discarded and the remaining sample, 300 µL supernatant including the pelleted material, was used for further extraction.
- Each sample was lysed by adding 20 µL proteinase K (Qiagen) to the vials followed by mix by vortex (Scientific Industries, Inc.), and lysis at 56 °C for 1 hour.
- The samples were extracted for total NA using the EZ1 RNA Tissue Mini Kit (Qiagen) according to manufacturer´s instructions. All extractions were performed on the EZ1 Advanced XL instrument (Qiagen), with an elution volume of 50 µL.
- After extraction, 15 uL of the total NA were reverse-transcribed to cDNA by using Illustra™ Ready-to-Go RT-PCR beads kit (GE Healthcare Life Science, Amersham, Place, UK) as previously described by Lager *et al.* (2017) [16]. The cDNA synthesis was prepared as single samples.
- A flowchart of the entire process is presented in Figure S7.

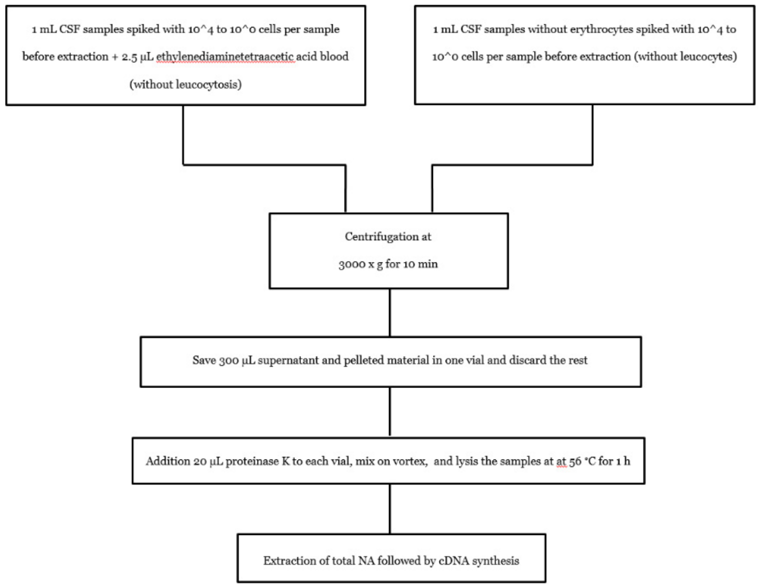

Figure S7: Flowchart for setup VI evaluating the influence of erythrocytes in the samples (CSF=cerebrospinal fluid, NA=nucleic acid, cDNA= complementary deoxyribonucleic acid)
